# Pseudolaric acid B ameliorates synovial inflammation and vessel formation by stabilizing PPARγ to inhibit NF‐κB signalling pathway

**DOI:** 10.1111/jcmm.16670

**Published:** 2021-06-11

**Authors:** Jiansen Lu, Hong Guan, Dan Wu, Zhiqiang Hu, Hongbo Zhang, Huaji Jiang, Jingyao Yu, Ke Zeng, Hongyu Li, Haiyan Zhang, Chenglong Pan, Daozhang Cai, Xiao Yu

**Affiliations:** ^1^ Department of Immunology School of Basic Medical Sciences Southern Medical University Guangzhou China; ^2^ Department of Orthopedics Orthopedic Hospital of Guangdong Province Academy of Orthopedics·Guangdong Province The Third Affiliated Hospital of Southern Medical University Guangzhou China; ^3^ Department of Joint Surgery the Fifth Affiliated Hospital of Southern Medical University Guangdong Province Guangzhou China; ^4^ The Third School of Clinical Medicine Southern Medical University Guangzhou China; ^5^ Guangdong Provincial Key Laboratory of Bone and Joint Degeneration Diseases Guangzhou China; ^6^ Department of Joint Surgery Center for Orthopaedic Surgery The Third Affiliated Hospital of Southern Medical University Guangzhou China; ^7^ School of Basic Medical Sciences Gannan Medical University Ganzhou China; ^8^ Guangdong Provincial Key Lab of Single Cell Technology and Application Southern Medical University Guangzhou China

**Keywords:** NF‐κB signalling, Osteoarthritis, PPARγ, pseudolaric acid B

## Abstract

Synovial macrophage polarization and inflammation are essential for osteoarthritis (OA) development, yet the molecular mechanisms and regulation responsible for the pathogenesis are still poorly understood. Here, we report that pseudolaric acid B (PAB) attenuated articular cartilage degeneration and synovitis during OA. PAB, a diterpene acid, specifically inhibited NF‐κB signalling and reduced the production of pro‐inflammatory cytokines, which further decreased M1 polarization and vessel formation. We further provide in vivo and in vitro evidences that PAB suppressed NF‐κB signalling by stabilizing PPARγ. Using PPARγ antagonist could abolish anti‐inflammatory effect of PAB and rescue the activation of NF‐κB signalling during OA. Our findings identify a previously unrecognized role of PAB in the regulation of OA and provide mechanisms by which PAB regulates NF‐κB signalling through PPARγ, which further suggest targeting synovial inflammation or inhibiting vessel formation at early stage could be an effective preventive strategy for OA.

## 1 INTRODUCTION

Osteoarthritis (OA), a damaging and chronic joint disease, is a dominant cause of chronic pain and joint dysfunction in the ageing adults, which essentially occurs in weight‐bearing joints.^1^, ^2^ Given that an increasing obese and ageing population, OA is more prevalent than before, which is characterized by graduated cartilage destruction, osteophyte formation, subchondral bone reconstruction, chronic synovial inflammation and vessel formation.^3^, ^4^ However, due to finite knowledge of its pathogenesis, there is no effective therapy to cure or prevent OA progression.

Recent studies showed that synovial inflammation was present in more than 40% of patients with symptomatic knee OA,^5^, ^6^ which is an increasing prominent feature during the progression of OA and is mainly caused by infiltration of activated macrophages.^7^, ^8^ During OA progression, lipopolysaccharide (LPS) acts as pathogen‐associated molecular patterns (PAMPs), while intra‐articular metabolites, including aggrecan, cartilage debris and fibronectin, serve as danger‐associated molecular patterns (DAMPs).^9^, ^10^ These PAMPs and DAMPs could be detected by pattern recognition receptors (PRRs) and initiate activation of NF‐κB, type I interferon and inflammasome pathways,^11^ releasing pro‐inflammatory cytokines and chemokines,^12^, ^13^ which classically activate synovial macrophages to polarize to M1 macrophages and secrete numerous pro‐inflammatory cytokines, including interleukin (IL)‐1, IL‐6 and tumour necrosis factor‐α (TNF‐α), thus leading to aggravating OA.^14^ Alternatively, macrophages could be polarized to M2 macrophages and secrete anti‐inflammatory cytokines, including IL‐4 and IL‐10, and ameliorate OA.^15^ Thus, regulating synovial macrophage polarization or anti‐inflammation treatment could be an effective strategy for the therapy and prevention of OA.

Pseudolaric acid B (PAB), one of the main components of Pseudolarix kaempferi Gordon (Pinaceae), is a diterpene acid with a compact tricyclic core molecular structure,^16^ indicating that PAB may have extensive biological functions, including anti‐angiogenesis,^17^ anti‐inflammatory and anti‐microbial activities.^16^, ^18^ PAB can prevent nuclear factor kappa‐light‐chain‐enhancer of activated B (NF‐κB) signalling in atherosclerosis progression.^19^ Recent study has uncovered that PAB has high affinity for peroxisome proliferator‐activated receptor γ (PPARγ),^20^ which further regulates NF‐κB signalling, inflammation and immune responses.^21^ In addition, PAB can prevent angiogenesis by promoting proteasome‐mediated degradation.^22^, ^23^ However, the distinct mechanisms interpreting potential contributions of PAB in macrophage polarization and anti‐inflammation in OA are largely unknown.

In this study, we demonstrated that PAB treatment decreased M1 macrophage polarization and inflammatory mediators in synovial macrophages during OA, which further attenuated vascular invasion and articular cartilage degeneration. PAB strongly inhibits NF‐κB‐dependent responses and attenuates the phosphorylation of p65 both in M1 macrophages and OA mice. Furthermore, we found that protective effects of PAB in OA synovium were achieved by inhibiting NF‐κB signalling through stabilizing PPARγ, and using PPARγ antagonist eliminates suppression of PAB on NF‐κB signalling. Taken together, our findings identify the protective function of PAB in OA progression and suggest that regulating synovial macrophage polarization or anti‐inflammation treatment could be useful therapeutic strategies for OA treatment.

## 2 MATERIALS AND METHODS

### 2.1 Materials

PAB (purity >98%) was purchased from Solarbio (Beijing, China), dissolved in 100% dimethyl sulphoxide (DMSO) (Sigma, St Louis, MO, USA). LPS and IL‐4 were obtained from Sigma‐Aldrich (St Louis, MO, USA) and dissolved in sterile water. Cell Counting Kit‐8 (CCK‐8) was obtained from Keygen Biotech (Nanjing, China). These antibodies were used in the study: anti‐MMP‐13 (1:200 for immunohistochemistry (IHC); Abcam, USA; ab39012), anti‐ColX (1:200 for IHC; Abcam, USA; ab58632), anti‐iNOS (1:50 for Immunofluorescence (IF); Abclone, Australia; A3200), HRP‐labelled goat anti‐rabbit IgG (H&L) (1:10 000 for western blots (WB), 1:100 for IHC; Jackson Immuno Research, USA; 111‐035‐003), goat anti‐rabbit IgG (H&L) Alexa Fluor 488 (1:200 for IF; Abcam; ab150077) and HRP‐labelled goat anti‐mouse IgG (H&L) (1:5000 for western blots; Jackson Immuno Research; 115‐035‐003).

### 2.2 Cells, cell culture and treatment

Raw 264.7 macrophages (ATCC, TIB‐71) were cultured in DMEM with high glucose (4.5 g/L; Gibco, USA), containing 10% fatal bovine serum (FBS) (Gibco, USA). For bone marrow (BM)‐derived macrophages (BMDMs), BM cells were obtained from the femur and tibia and incubated in 10%‐20% L929 conditioned media with macrophage colony‐stimulating factor (M‐CSF) (St Louis, MO, USA) for 6 days, as previously described.^24^, ^25^ The pre‐chondrocyte cell line ATDC5 (Tsukuba, Japan) was cultured in DMEM/F12 (Gibco, USA), containing 1×ITS (BD Biosciences) and 5% FBS, as previously described.^26^ All cells were cultured at 37°C with 5% CO_2_, and the medium was replaced every 1‐2 days. For M1/M2 macrophage polarization, Raw 264.7 and BMDM cells were administrated with LPS (100 ng/mL) for 12 hours to induce M1‐like macrophages and IL‐4 for 24 hours (20 ng/mL) to gain M2‐like macrophages. PAB (0.75 and 1.5 μmol/L) was administered in cells for 12 hours. T0070907 (10 μmol/L, Sigma, St Louis, MO, USA; T8703) was co‐treated with PAB in cells for 12 hours. And cells were administrated with 0.1% DMSO acted as the control. Supernatant samples were further collected and subjected to ELISA analysis.

### 2.3 Cell Viability Assay

Cell viability was detected by the CCK‐8 assay. Raw 264.7 cells were cultured in 96‐well plates (5 × 10^3^ cells/cm^2^) for 24 hours. Briefly, Raw 264.7 cells were pretreated with or without several concentrations (0, 0.3, 0.75, 1.5, 3.0 and 6.0 μmol/L) of PAB for 12 hours. After washing the cells, DMEM containing 10% CCK‐8 solution was incubated at 37°C. Absorbance was analysed at 450 nm by the Multiskan FC (Thermo Fisher, Waltham, MA, USA).

### 2.4 Animals, OA model and treatment

Animal experiments were authorized by the Southern Medical University Animal Care and Use Committee (SMUL2021014). Forty 8‐week‐old male C57/BL6 mice (24‐30 g) were acquired from the Experimental Animal Centre of Southern Medical University. Animals were subjected to surgery destabilizing the medial meniscus (DMM) on the right knees after anaesthetized with isoflurane (Solarbio, Beijing, China) as previously described.^26^ Briefly, the incision of skin and joint capsule in the right knees was performed, and the ligaments of the medial meniscus were disconnected by microsurgical scissors, leading to knee joint instability and then inducing post‐traumatic OA (n = 30). The sham operation was only incision of the skin and joint capsule and the wound was stitched (n = 10). Beginning at 1 day after DMM surgery, experimental mice from the DMM group were administered with PAB (Stock solution dissolved in saline, 5 or 10 mg/kg, n = 20), while control mice were treated with vehicle by intra‐articular injection twice a week for 5 or 10 weeks (n = 10). 5 or 10 weeks after surgery, all mice were killed to collect the right knee joints (Figure S1A).

### 2.5 Preparation of paraffin‐embedded specimens, histochemistry, immunohistochemistry and immunostaining

Dissected mouse right knees were soaked in 4% paraformaldehyde (PFA, Sigma‐Aldrich, St Louis, MO, USA) for 48 hours. After decalcification, joints were embedded in paraffin, and then the specimens were performed sections continuously with 4 μm thick. As previously described, Safranin‐O/Fast Green staining was carried out.^27^ Articular cartilage destruction on tibia was analysed by the Osteoarthritis Research Society International (OARSI) scoring system. As previously described, H&E was performed to assess synovitis by counting synovial lining cell thickness (0‐3),^28^, ^29^ and the sum of lateral and medial compartments of the joint is shown (0‐6). For IF and IHC, sections were incubated with sodium citrate repair solution (Sigma‐Aldrich, St Louis, MO, USA) to unmask antigens. For IF, sections were incubated with 10% sheep serum and then soaked with primary antibodies at 4°C overnight. After washing three times, antibodies labelled with Alexa Fluor 488 were used, and nuclei were identified with 4′,6‐diamidino‐2‐phenylindole (Thermo, MA, USA) before imaging, as previously described.^27^ For IHC, sections were also administered with 10% sheep serum and incubated with indicated antibodies after soaking in 3% hydrogen peroxide solution. Chromogen was observed by 3,3′‐diaminobenzidine, and then hematoxylin staining was performed.

### 2.6 ELISA

Cell supernatants and serum were detected by mouse IL‐1β, IL‐6 and TNF‐α ELISA kit (#E‐EL‐M0037c, #E‐EL‐M0044c, #E‐EL‐M1084c; Elabscience Biotechnology). Absorbance was detected at 450 nm by the Multiskan FC (Thermo Fisher, Waltham, MA, USA).

### 2.7 Immunoblot analysis

Lysis buffer was prepared with 10 mmol/L Tris‐HCl (pH 6.8), 10 mmol/L dithiothreitol, 10% glycerol, 1 mmol/L phenylmethylsulphonyl fluoride, 2% sodium dodecyl sulphate and 10% β‐mercaptoethanol. Cells and tissues were lysed and separated with SDS‐PAGE, blotted onto PVDF membranes (Sigma‐Aldrich, St Louis, MO, USA) and blocked with 5% milk. Then, the samples were incubated with indicated antibodies overnight. For all blots, EMD Millipore Luminata Western HRP Chemiluminescence Substrate was used for protein detection.

### 2.8 Quantitative reverse transcription‐polymerase chain reaction

The total RNA was purified from tissues or cells by TRIzol reagent (Invitrogen, Thermo Fisher Scientific, Waltham, MA, USA), as previously described.^27^ Then, cDNA was reverse transcribed by StarScript II first‐strand cDNA synthesis kit (Genstar, Beijing, China), and PCR was performed with 2x RealStar green power mixture (Genstar, Beijing, China) on QuantStudio 6 flex (Thermo Fisher, Waltham, MA, USA) with the following primers:

*Col2a1*forward: CACACTGGTAAGTGGGGCAAGACCG

*Col2a1* reverse: GGATTGTGTTGTTTCAGGGTTCGGG

*Sox9* forward: TACCTACGGCATCAGCAGCTC

*Sox9* reverse: TTGCCTTCACGTGGCTTTAAG

*inos* forward: GGAGTGACGGCAAACATGACT

*inos* reverse: TCGATGCACAACTGGGTGAAC

*Il6* forward: CTCTGGGAAATCGTGGAAAT

*Il6* reverse: CCAGTTTGGTAGCATCCATC

*Il1b* forward: GCAACTGTTCCTGAACTCAACT

*Il1b* reverse: GTGCTCATGTCCTCATCCTG

*Tnfa* forward: GACGTGGAACTGGCAGAAGAG

*Tnfa* reverse: TTGGTGGTTTGTGAGTGTGAG

*Colx* forward: AAAGCTTACCCAGCAGTAGG

*Colx* reverse: ACGTACTCAGAGGAGTAGAG

*Mmp13* forward: CTTCTTCTTGTTGAGCTGGACTC

*Mmp13* reverse: CTGTGGAGGTCACTGTAGACT

*Runx2* forward: TCCCCGGGAACCAAGAAGGCA

*Runx2* reverse: AGGGAGGGCCGTGGGTTCTG

*Gapdh* forward: AGGTCGGTGTGAACGGATTTG

*Gapdh* reverse: TGTAGACCATGTAGTTGAGGTCA

### 2.9 Statistical analysis

All experiments were repeated at least three times. And data were represented as mean ± SD by statistical product and service solutions (SPSS) version 21.0 software (USA). Statistically significant differences in each group were evaluated using two‐tailed Student’s *t* test. Two‐tailed Student's *t* test was evaluated statistically significant differences in each group. And curve data were analysed by GraphPad Prism 8.0 (USA). The statistical significance was defined as *P* < .05.

## 3 RESULTS

### 3.1 PAB attenuates OA by decreasing loss of cartilage and synovial inflammation

**FIGURE 1:**
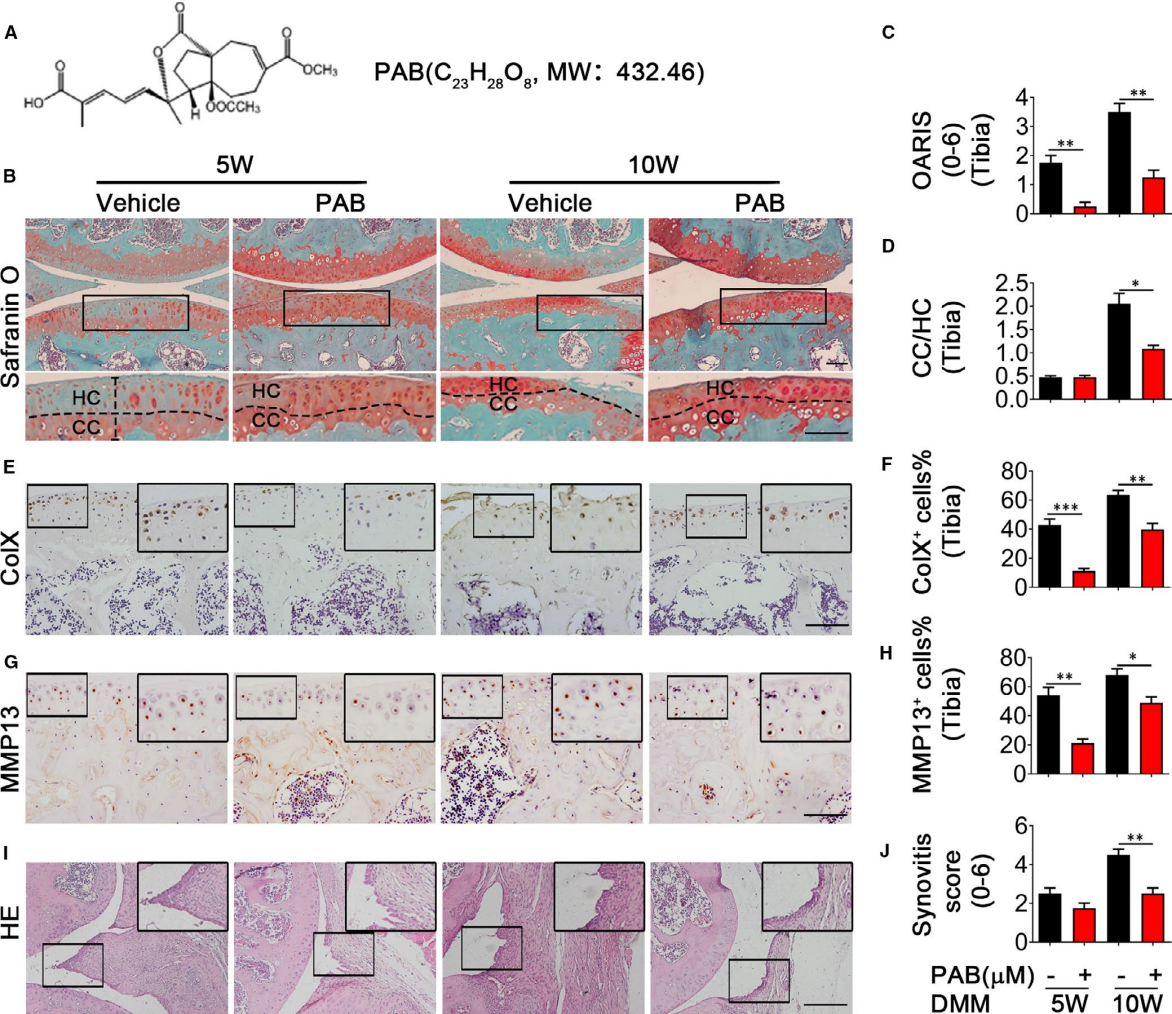
PAB attenuates cartilage damage and synovitis in DMM mice. A, Chemical structure of PAB. B, Cartilage degradation evaluated by Safranin O and Fast Green staining. Higher magnification is shown at the bottom of panel B. Dotted lines demonstrate tide line. Scale bar: 50 µm. C, OARSI score was assessed in CIOA mice treated with vehicle or PAB. D, Cartilage thickness was assessed using ratio between calcified cartilage (CC) and hyaline cartilage (HC). E‐H, Immunostaining and quantitative analysis of cells positive for ColX and MMP13 in vehicle or PAB‐treated DMM mice at five or ten weeks after surgery. I, H&E staining in DMM mice treated with vehicle or PAB. Scale bar: 50 µm. Higher magnification is demonstrated on the right top. J, Synovitis score quantification in the synovium of vehicle or PAB‐treated DMM mice (n ≥ 4). DMM, destabilizing the medial meniscus; PAB, Pseudolaric acid B; ColX, collagen X; MMP13, matrix metallopeptidase 13. *P < .05, **P < .01, ***P < .001

To determine the effects of PAB in OA, we generated a model by intra‐articular injection of PAB in destabilizing the medial meniscus (DMM) mice (Figure S1A) and used 10 mg/kg PAB in the following animal experiments through analysis of PAB’s effect at different concentrations (5 or 10 mg/kg) (Figure S1B). The molecular structure of PAB was demonstrated in Figure 1A. And we found that mice treated with PAB demonstrated significantly reduced cartilage destruction (Figure 1B) and lower OARSI score (Figure 1C) at both 5 and 10 weeks after operation. Meanwhile, hyaline cartilage thickness maintained well in PAB‐treated DMM mice at 10 weeks after surgery, compared to increased calcified cartilage thickness in DMM mice treated with vehicle (Figure 1D). Furthermore, chondrocyte hypertrophic differentiation markers (collagen X (ColX) and matrix metallopeptidase (MMP) 13) reduced in PAB‐treated mice (Figure 1E–H), indicating that PAB prevented the development of post‐traumatic OA in vivo. Increasing evidence supports that low‐grade inflammation functions as an important mediator of the pathogenesis of OA, which may contribute to cartilage destruction, serious joint symptoms, movement disorder and radiographic grades.^5^ Indeed, knee synovitis was also detected in both groups. Although PAB treatment showed a slight reduction in synovial hyperplasia, there are no significant differences in synovitis score between PAB‐treated and control mice at 5 weeks after operation. However, knee synovitis score was dramatically decreased in PAB‐treated mice at 10 weeks after DMM surgery (Figure 1I, J), indicating that early inflammation may be complex and needs a continuous treatment. Collectively, these data indicate that PAB prevents the development of post‐traumatic OA by inhibiting synovial inflammation in vivo.

### 3.2 PAB decreases M1 macrophage polarization and inhibits inflammatory cytokines in vitro and in vivo

**FIGURE 2:**
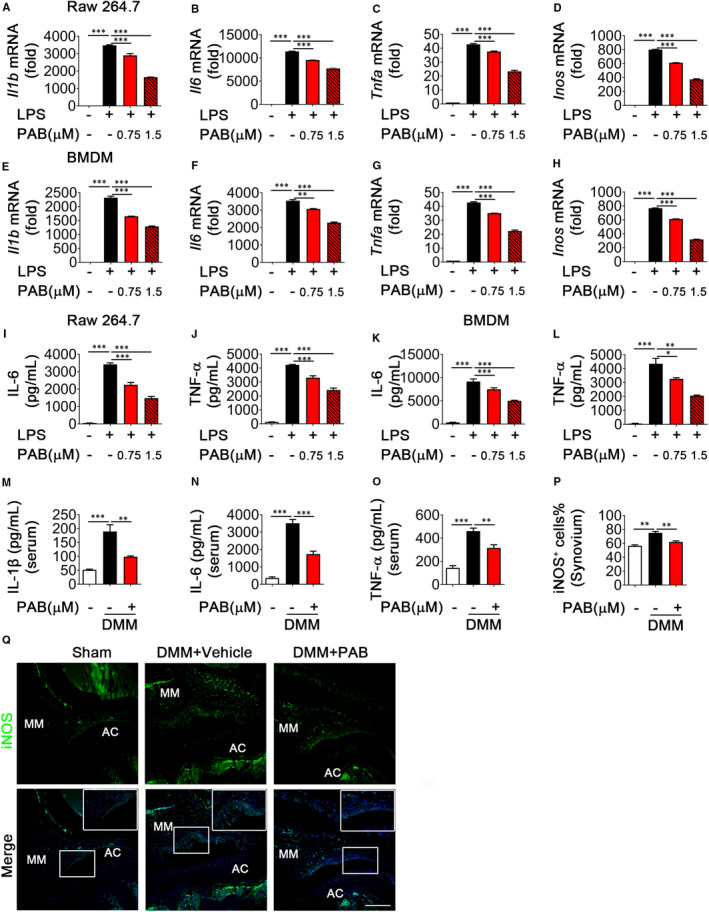
PAB decreases inflammatory cytokines during M1 macrophage polarization in vitro and in vivo. A‐H, Quantitative PCR analysis of IL‐1β (A and E), IL‐6 (B and F), TNF‐α (C and G) and iNOS (D and H) expression in LPS‐induced Raw 264.7 (A–D) and BMDM cells (E–H) treated with or without PAB. I–L, ELISA analysis of IL‐6 (I and K) and TNF‐α (J and L) levels in the supernatant of Raw 264.7 (I and J) and BMDM (K and L) cells treated with LPS and co‐treated with PAB or vehicle (n = 3). (M–O) ELISA analysis of IL‐1β (M), IL‐6 (N) and TNF‐α (O) level in the serum of vehicle or PAB‐treated DMM mice at five weeks after surgery. (P and Q) Immunostaining and quantitative analysis of iNOS^+^ cells in vehicle or PAB‐treated DMM mice at five weeks after surgery (n ≥ 4). Scale bar: 50 µm. Higher magnification is demonstrated on the right top. Sham, sham surgery; AC, articular cartilage; MM, medial meniscus. *P < .05, **P < .01, ***P < .001

Our previous study showed that M1‐polarized macrophages were associated with synovial inflammation during OA.^26^ We next sought to determine whether PAB regulates macrophage polarization and production of cytokines during synovial inflammation. Dose titration of PAB on the viability of Raw 264.7 (Figure S2A) and BMDM (Figure S2B) cells was performed by CCK‐8 assay, and 0.75 and 1.5 μmol/L of PAB was used for the subsequent experiments. We first detected the potential role of PAB on LPS‐induced inflammatory reactions in Raw 264.7 cells and found LPS‐induced production of IL‐1β, IL‐6, TNF‐α and iNOS was diminished by PAB treatment in a dose‐dependent manner (0.75 and 1.5 μmol/L) in Raw 264.7 cells (Figure 2A‐D). Similar results were observed in BMDM cells (Figure 2E‐H). Consistently, the expression of IL‐6 and TNF‐α in the supernatants was inhibited by PAB treatment in LPS‐induced Raw 264.7 (Figure 2I, J) and BMDM cells (Figure 2K, L). Since the release of IL‐1β in the supernatant is more complex, which needs ATP, ROS or DNA‐induced inflammasome cleave pro‐IL‐1β to mature IL‐1β and then release IL‐1β to the supernatants, ^30^ we detected IL‐1β protein level after LPS stimulation and ATP treatment and found the release of IL‐1β was inhibited by PAB treatment in Raw 264.7 and BMDM cells (Figure S2C, D). Moreover, we also determined the potential role of PAB in M2‐like macrophage induced by IL‐4. The expression levels of CD206 and IL‐10 increased in PAB‐treated Raw 264.7 (Figure S2E, F) and BMDM cells (Figure S2G, H), indicating that PAB regulates macrophage reprogramming from M1 to M2 subtype. Furthermore, inflammatory factors (IL‐1β, IL‐6 and TNF‐α) decreased in the serum of PAB‐treated DMM mice at 5 weeks after surgery, compared to vehicle‐treated DMM mice (Figure 2M‐O), and percentage of iNOS^+^ cells (M1 macrophage polarization) also decreased after PAB treatment (Figure 2P, Q). These data indicate that PAB decreases M1 macrophage polarization and inflammatory cytokines in vitro and in vivo.

### 3.3 PAB prevents vessel formation in vitro and in vivo

**FIGURE 3:**
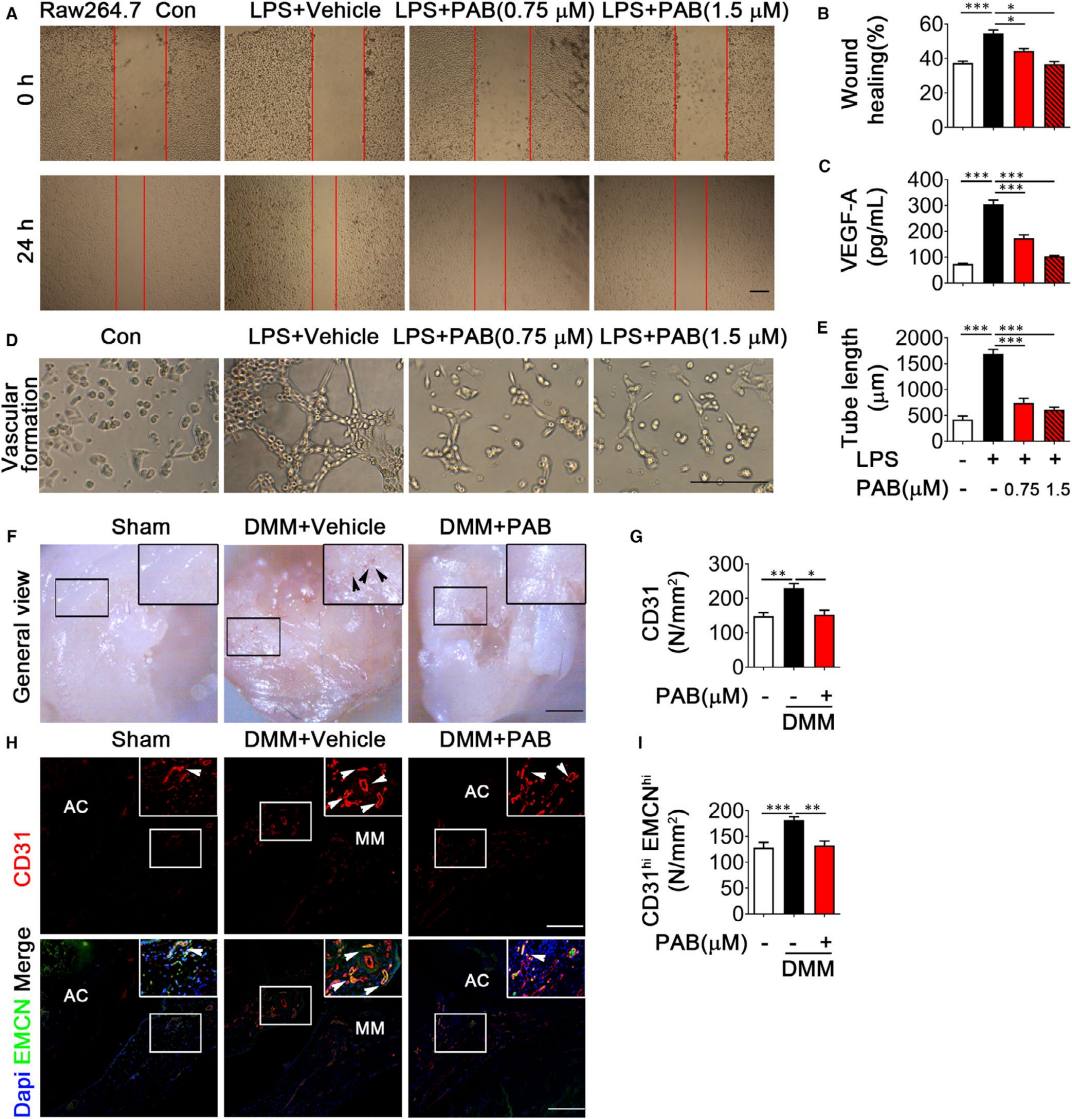
PAB prevents vessel formation in vitro and in vivo. A, B, HUVECs were co‐cultured with CM, vehicle‐treated M1 macrophage CM or PAB‐treated M1 macrophage CM from LPS‐induced Raw 264.7 cells for 24 h. Wound healing assay was performed. Scale bar: 50 µm. C, VEGF‐A levels in the supernatant of LPS‐stimulated Raw 264.7 cells treated with or without PAB were analysed by ELISA. D, E, HUVECs were co‐cultured with the supernatant of Raw 264.7 cells, and tube formation was evaluated by tube formation assay (D). HUVEC tube length was measured and demonstrated in (E). Scale bar: 50 µm. (n = 3). F, Tibias from sham, vehicle‐treated DMM mice and PAB‐treated DMM mice were presented after 5 wk surgery. G‐I, Representative immunofluorescence double staining (H) and quantification (G and I) of cells positive for endomucin (green) and CD31 (red) in mice after DMM surgery for 5 weeks (n ≥ 4), mice were treated with or without PAB as shown. Scale bar: 50 µm. Higher magnification is demonstrated on the right top. Sham, sham surgery; AC, articular cartilage; MM, medial meniscus. CM, conditioned medium. *P < .05; **P < .01; ***P <.001

Since synovial inflammation is accompanied with vessel formation at early stage of OA,^2^ accumulating evidence shows that inflammatory cytokines can promote vessel formation during OA.^31^ Moreover, recent studies showed that PAB inhibits angiogenesis in some tumour models. To determine whether PAB affects vessel formation during OA, we obtained conditioned medium (CM) from the M1‐like macrophages treated with or without PAB and then co‐cultured the CM with human umbilical vein endothelial cells (HUVECs). As expected, we found PAB can inhibit HUVECs migration, and PAB‐treated CM‐co‐cultured HUVECs showed poor wound healing (Figure 3A, B). VEGF‐A is an increasing vital pro‐angiogenic mediator during OA and the expression levels of VEGF‐A were inhibited by PAB treatment (Figure 3C). Moreover, CM from PAB‐treated M1‐like macrophage inhibited tube formation in vitro, compared with the CM from the M1‐like macrophage treated without PAB (Figure 3D, E). Consistent results were observed in BMDM cells (Figure S3A‐E). Furthermore, we observed more vessels in DMM mice treated with vehicle, and less vessels in PAB‐treated DMM mice and sham mice at five weeks post‐surgery (Figure 3F). Strong CD31 immunostaining was shown in DMM mice but not in the sham or PAB‐treated DMM mice (Figure 3G, H). Recent study indicated that H‐type vessel, which highly expresses CD31 and endomucin (CD31^hi^Emcn^hi^), is a specific vessel subtype, which is accompanied by osteogenesis.^32^ Importantly, our previous study demonstrated H‐type vessels enhanced in DMM mice.^33^ To investigate whether PAB could decrease H‐type vessels in the synovium during OA, we investigated double‐positive endomucin and CD31 cells in a OA model by immunofluorescence staining and observed H‐type vessels markedly increased in the synovium of OA mice, and decreased in PAB‐treated OA mice (Figure 3H, I).

Mechanistic target of rapamycin (mTOR) is a crucial mediator of metabolism, proliferation and apoptosis in response to stimulus, including growth factors and nutrients,^34^ and excessive nutrition (vessels) can exacerbate OA progression by enhancing inflammatory cell infiltration.^35^ To detect whether PAB affects the crosstalk between inflammation and angiogenesis, cells were starved by serum deprivation and then restored with nutrition, which could activate mTOR signalling pathways. We observed that inflammatory cytokines slightly reduced in starved Raw 264.7 cells, and restored nutrition could increase the production of inflammatory cytokines and phosphorylation of S6 by mTOR activation (Figure S3F‐K). Furthermore, we found phosphorylation of S6 in both cartilage and synovium was inhibited by PAB treatment in DMM mice, compared to vehicle‐treated DMM mice (Figure S3L‐N), suggesting that PAB can inhibit H‐type vessels by down‐regulating mTOR signalling pathways. Taken together, these findings suggest that PAB alleviates OA progression partially by blocking the positive feedback between inflammation and angiogenesis.

### 3.4 PAB inhibits NF‐κB signalling activation during OA

**FIGURE 4:**
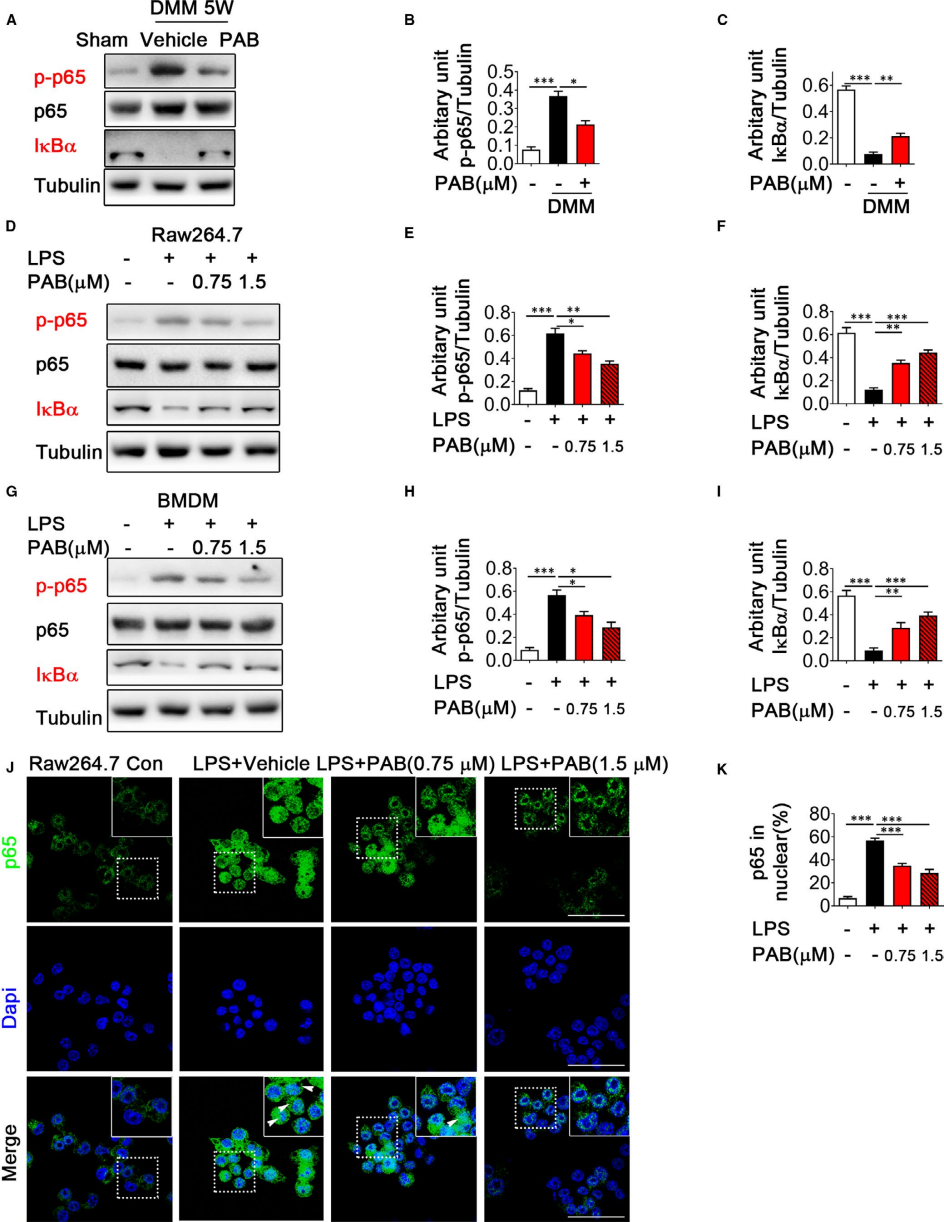
PAB attenuates NF‐κB activation during OA in vivo and in vitro. A‐C, Immunoblot analysis (A) of p‐p65 and IκBα expression in the synovium of vehicle or PAB‐treated DMM mice at five weeks after surgery. Analysis of grey intensity was shown in B and C. D‐F, Immunoblot analysis of p‐p65 and IκBα expression in LPS‐induced Raw 264.7 cells treated with or without PAB was shown in D, analysis of grey intensity was shown in E and F. G‐I, Immunoblot analysis of p‐p65 and IκBα expression in LPS‐induced BMDM treated with or without PAB was shown in G, analysis of grey intensity was shown in H and I. J, K, Raw 264.7 cells were treated with LPS (1 μg/mL) for 30 min after treated with vehicle or PAB for 3h. The nucleus was stained by DAPI (blue) and the localization of p65 (green) was determined by immunostaining in (J). Scale bar: 50 μm. Higher magnification is demonstrated on the right top. Percentage of nuclear localized cells for p65 was shown in (K). *P <.05, **P < .01, ***P < .001

Next, we sought to determine the molecular mechanisms by which PAB prevents OA. Since recent studies showed that PAB ameliorates atherosclerosis progression by blocking NF‐κB activation,^19^ we hypothesised that PAB regulates OA progression and synovial inflammation by targeting NF‐κB signalling. We compared the activation of NF‐κB signalling in the OA synovial tissue of mice treated with vehicle or PAB at 5 weeks after surgery and observed that NF‐κB signalling pathway was up‐regulated in OA mice. However, phosphorylation of p65 and degradation of IκBα were suppressed in the synovium of PAB‐treated DMM mice (Figure 4A‐C), which suggest PAB treatment inhibits NF‐κB signalling during OA progression. Moreover, we investigated how PAB inhibits inflammation in LPS‐stimulated Raw 264.7 and BMDM cells. Similarly, LPS‐induced phosphorylation of p65 was inhibited, while degradation of IκBα was restored by PAB treatment in Raw 264.7 (Figure 4D‐F) and BMDM cells (Figure 4G‐I). Furthermore, immunofluorescence analysis revealed that PAB greatly antagonized the process of NF‐κB p65 nuclear translocation in Raw 264.7 cells (Figure 4J, K). Therefore, these data indicate that PAB inhibits NF‐κB activation in macrophages during OA.

### 3.5 PAB inhibits NF‐κB pathway by stabilizing PPARγ during OA

**FIGURE 5:**
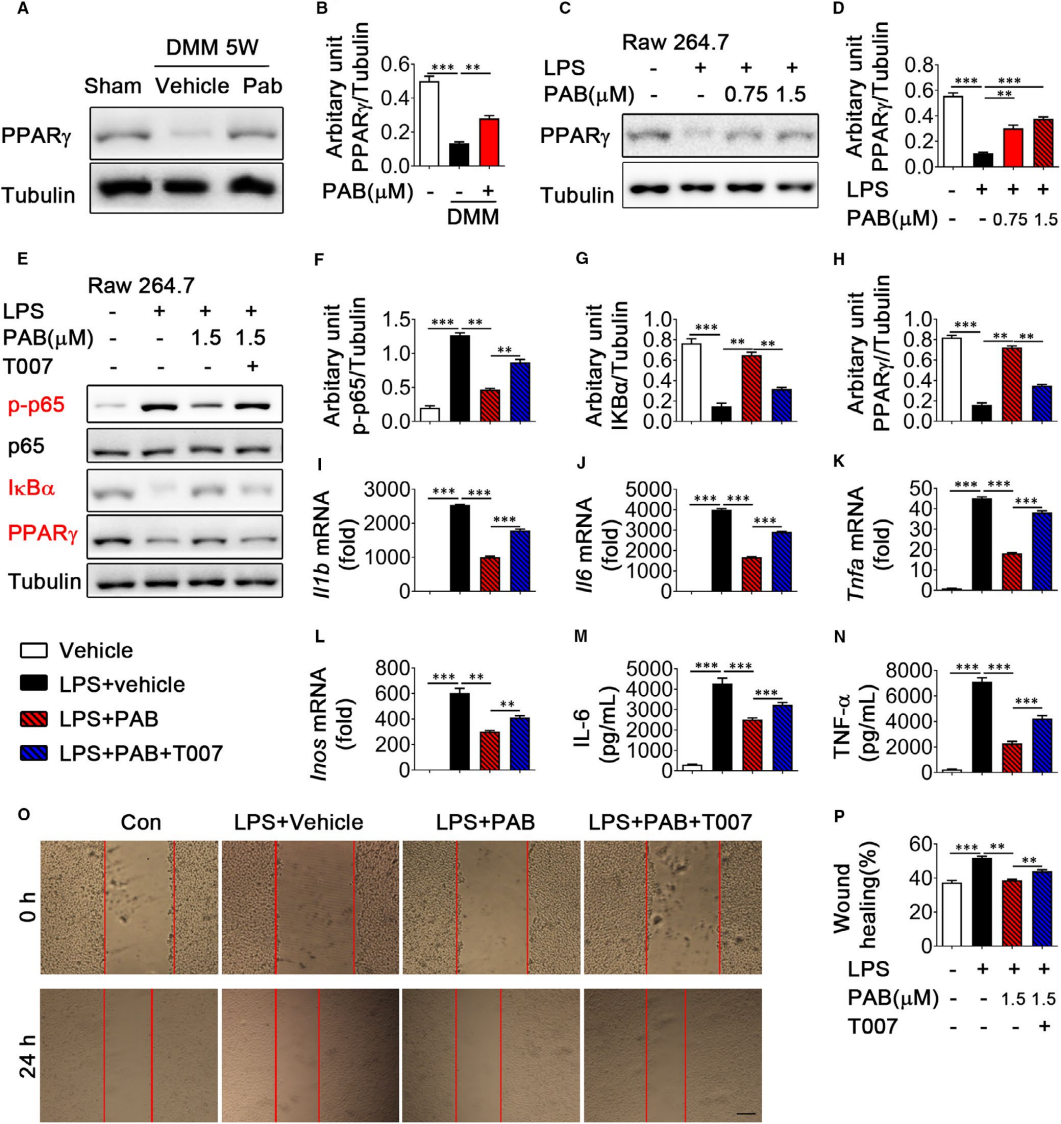
PAB inhibits NF‐κB pathway by enhancing PPARγ expression in OA mice and LPS‐induced Raw 264.7 cells. A,B, DMM mice were treated with or without PAB. Immunoblot analysis of PPARγ expression in the synovium of vehicle‐ or PAB‐treated DMM mice at five weeks after surgery (A), and analysis of grey intensity was shown in (B) (n ≥ 4). C, D, LPS‐induced Raw 264.7 cells were treated with or without PAB, the expression level of PPARγ was shown in (C) and analysis of grey intensity was shown in (D). E‐H, LPS‐induced Raw 264.7 cells were treated vehicle, PAB or PAB plus T0070907 (T007, PPARγ antagonist). Immunoblot analysis of p‐p65, IκBα and PPARγ was shown in (E), analysis of grey intensity was shown in (F‐H). I‐L, Quantitative PCR analysis of IL‐1β (I), IL‐6 (J), TNF‐α (K) and iNOS (L) in LPS‐induced Raw 264.7 cells treated with vehicle, PAB or PAB plus T0070907. M,N, ELISA analysis of IL‐6 (M) and TNF‐α (N) levels in the supernatant of LPS‐induced Raw 264.7 cells treated with vehicle, PAB or PAB plus T0070907. O,P, LPS‐induced Raw 264.7 cells were treated vehicle, PAB or PAB plus T0070907. Wound healing assay was performed. (n = 3) Scale bar: 50 µm. Raw 264.7 cells were treated vehicle (Con), and LPS‐induced Raw 264.7 cells were treated vehicle (LPS+vehicle), PAB (LPS+PAB) or PAB plus T0070907 (LPS+PAB+T007). *P < .05, **P < .01, ***P < .001

Since PPARγ can block NF‐κB activation by increasing the expression of phosphatase and tensin homolog (PTEN), IκBα and sirtuin 1 during the inflammatory reaction,^36^ and PAB has high affinity for PPARγ,^21^ we thus speculated that PAB regulates activation of NF‐κB signalling through mediating expression of PPARγ. Our results demonstrated that expression of PPARγ was diminished in DMM mice and restored by PAB treatment during OA progression (Figure 5A, B). And similar results were observed in LPS‐stimulated Raw 264.7 cells treated with PAB (Figure 5C, D). To further confirm that PAB inhibits NF‐κB signalling by stabilizing PPARγ, we used PPARγ antagonist T0070907 (T007 for short) to reduce PPARγ expression in LPS‐stimulated Raw 264.7 cells and found PAB treatment could not suppress phosphorylation of p65 and restore the degradation of IκBα while PPARγ expression is inhibited by T0070907 in LPS‐stimulated Raw 264.7 cells (Figure 5E‐H). As expected, q‐PCR analysis showed the expression of IL‐1β, IL‐6, TNF‐α and iNOS increased in LPS‐, T0070907‐ and PAB‐treatment group, compared with the LPS‐ and PAB‐treatment group (Figure 5I‐L). Meanwhile, release of IL‐6 and TNF‐α also enhanced in the supernatant of T0070907 and PAB co‐treated cells (Figure 5M, N). Furthermore, T0070907 rescued the effect of PAB in HUVECs migration (Figure 5O, P). Together, these findings indicate that PAB regulates NF‐κB signalling and decreases inflammatory cytokines by stabilizing PPARγ during OA.

### 3.6 PAB attenuates cartilage degeneration by regulating PPARγ in OA

**FIGURE 6:**
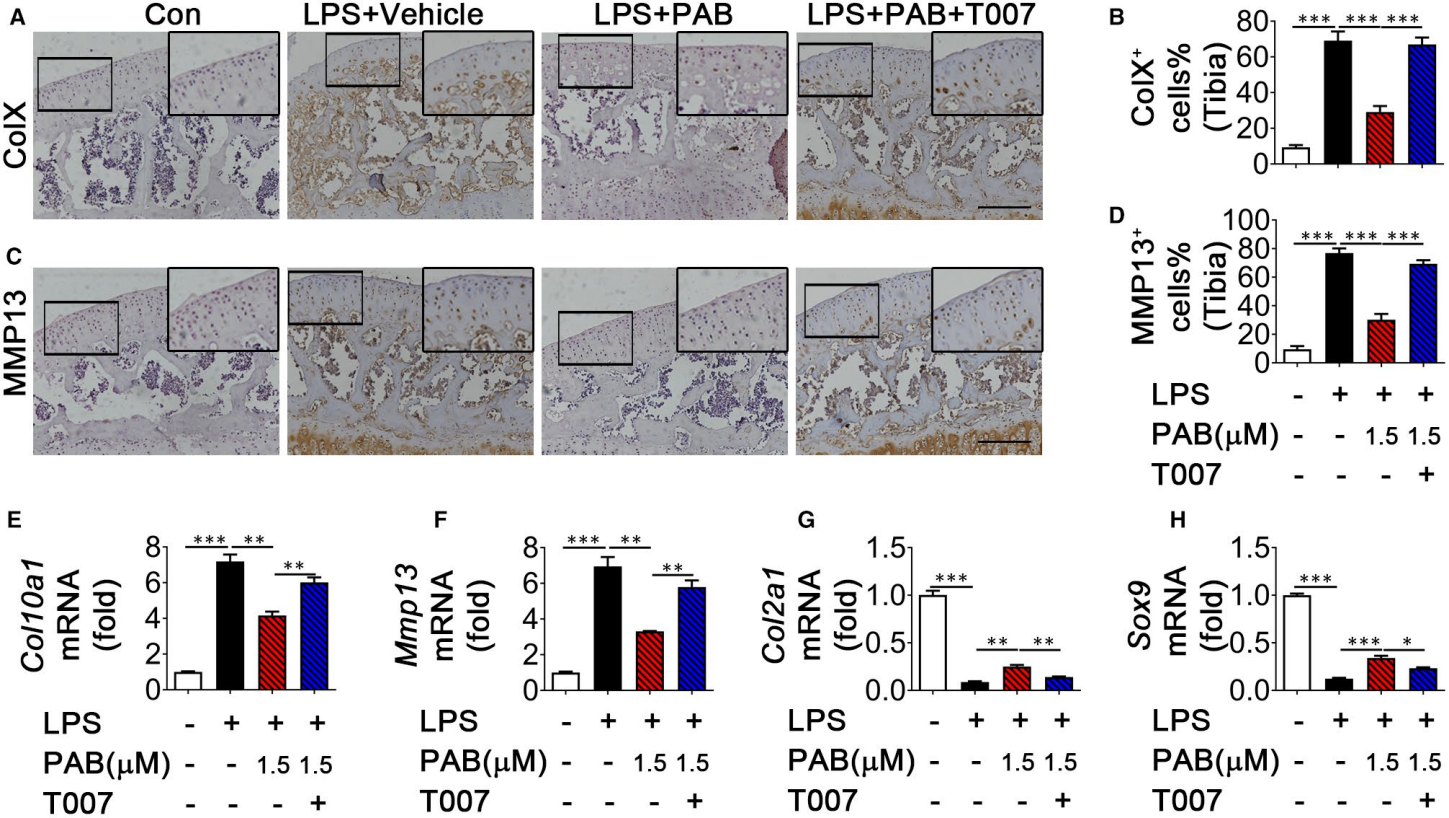
PAB prevents cartilage degeneration by regulating PPARγ expression during OA. Cartilage explants were co‐cultured with CM, vehicle‐treated M1 macrophage CM, PAB‐treated M1 macrophage CM or PAB plus T0070907‐treated M1 macrophage CM for 72 h. (A‐D) Immunostaining and quantitative analysis of cells positive for ColX (A and B) and MMP13 (C and D) in cartilage explants co‐cultured as shown (n = 3), scale bar: 50 µm. Higher magnification is demonstrated on the right top. Scale bar: 100 µm. (E‐H) ATDC5 were co‐cultured with CM, vehicle‐treated M1 macrophage CM, PAB‐treated M1 macrophage CM or PAB plus T0070907‐treated M1 macrophage CM for 24 h. Quantitative PCR analysis of Col10a1 (E), MMP13 (F), Col2a1(G) and Sox9 (H) in ATDC5. ColX, collagen X; MMP13, matrix metallopeptidase 13; Sox9, SRY‐related HMG box‐containing SOX 9. *P < .05, **P < .01, ***P < .001

Finally, to determine whether PAB regulates OA progression and cartilage degeneration through stabilizing PPARγ, we cultured cartilage explants with CM from the M1‐like macrophage treated with or without T0070907 and found protection of PAB on OA progression was diminished when using PPARγ antagonist T0070907. Moreover, immunohistochemistry analysis showed that ColX and MMP13 expression levels were restored by T0070907 co‐treatment as compared with the explants treated with LPS and PAB alone (Figure 6A‐D). Meanwhile, T0070907 enhanced ColX and MMP13 levels in ATDC5 co‐cultured with PAB‐treated M1 macrophage CM (Figure 6E, F). Furthermore, T0070907 can reduce expression of specific chondrogenic markers (Col2a1 and SRY‐related HMG box‐containing (SOX) 9) in ATDC5 co‐cultured with M1 macrophage CM treated with PAB (Figure 6G, H). Together, these findings indicate that PAB restrains chondrocyte catabolism and ameliorates cartilage destruction *via* regulating PPARγ expression.

## 4 DISCUSSION

OA is a systemic disease, including the involvement of cartilage degeneration, bone remodelling and chronic synovial inflammation. Synovial inflammation and angiogenesis are critical in the pathogenesis of OA. This study suggested an important function of PAB in the pathogenesis and progression of OA. Locally released DAMP activates NF‐κB signalling and results in production of pro‐inflammatory cytokines, which results in M1 macrophage polarization and articular cartilage degeneration during OA progression. PAB specifically targets and stabilizes PPARγ to inhibit NF‐κB signalling in synovial tissues, decreases pro‐inflammatory cytokines production and suppresses M1 macrophage polarization, synovial inflammation and vessel formation, which consequently ameliorates articular cartilage degeneration during OA (Figure S4). Thus, our findings suggest that PAB treatment by intra‐articular injection may offer a new strategy to block synovial inflammation and angiogenesis during OA.

Synovitis is an increasing character in OA^7^ and accumulating evidences showed that persistent low grade of synovial inflammation plays a key role in OA progression.^5^ During synovial inflammation, activated synovial macrophages polarize to the M1 phenotype in response to pathological stresses and joint injury,^37^ which accumulate multitudinous inflammatory cytokines, then leading to chronic OA pain.^14^ Consistently, our previous study demonstrated that M1‐polarized macrophages increased in mouse and human OA synovium.^26^ The pre‐existing systemic inflammation emerges symptoms similar to RA synovitis in the unhurt joint and accelerated post‐traumatic OA progression after injury.^38^ Furthermore, blocking inflammatory factors attenuates cartilage degeneration during OA.^39^, ^40^ Therefore, non‐steroidal anti‐inflammatory drugs (NSAIDs) are the most common used clinical prescriptions to alleviate OA pain. However, long‐term use of NSAIDs could be associated with multiple complications, including acute renal impairment and dyspepsia.^41^ Thus, an effective therapy is urgently needed as an alternative to take the place of NSAIDs in curing OA. PAB, containing a compact tricyclic core molecular structure, maintains anti‐inflammatory effects in many diseases, including atherosclerosis and skin lesions.^19^, ^42^ Here, we observed that PAB decreased M1‐polarized macrophages in vivo and in vitro, leading to decreasing synovitis, which suggest PAB could be an potential drug applicable in relieving synovial inflammation during OA.

Alternatively, synovial macrophages can be also polarized to the M2 phenotype during anti‐inflammatory reaction, which release lots of anti‐inflammatory mediators.^15^ The imbalance of M1/M2‐polarized macrophages is also involved in OA.^43^ Thus, regulating M1/M2 subtype ratio may have a protective effect on OA progression. Accumulating evidences highlighted that switching the polarization from M1 to M2 subtypes prevents synovitis during OA progression.^44^ Here our results showed PAB decreased M1‐polarized macrophages and enhanced M2‐polarized macrophages, which further suggest that targeting synovial inflammation may attenuate OA progression partially by regulating M1/M2 subtype ratio.

Synovial inflammation and vessel formation are both observed in early OA.^2^ Recent studies showed that most of inflammatory cytokines can accelerate angiogenesis in OA.^31^ Indeed, we also found that inflammatory cytokines stimulated vessel formation in vivo and in vitro. PAB blocks angiogenesis and decreases hypoxia‐inducible factor 1 alpha in some cancer diseases.^21^ Here we observed that PAB decreased inflammatory cytokines and blocked VEGF‐A levels in LPS‐induced supernatants, which further inhibited tube formation in vitro, indicating that PAB could inhibit angiogenesis during OA. H‐type vessel, a specific subtype, is closely related to osteogenesis.^32^ We previous reported that H‐type vessels accumulated in the knee joints of OA mice and blocking H‐type vessels decreased loss of cartilages during OA.^33^ Here we further identified PAB is responsible for blocking H‐type vessels in synovium during OA.

The new blood vessels provide oxygen and nutrients,^45^ which could activate mTORC1 signalling and transport more inflammatory mediators, and also contribute to the persistence of inflammation.^46^ Here, we observed that M1 macrophage CM presented pronounced accumulation of inflammatory cytokines and starvation with low mTORC1 activation showed reduction of inflammatory cytokines in Raw 264.7 cells. Meanwhile, enhanced inflammatory cells secrete many pro‐angiogenic mediators and facilitate subsequently neo‐vascular invasion, which establish the positive feedback between inflammation and angiogenesis.^46^ Blocking the positive feedback regulation of inflammation and vessel may alleviate OA development. Thus, it is suggested that new treatments for OA are needed that not only target synovial inflammation, but also selectively prevent angiogenesis.^47^ Our present study demonstrated that PAB alleviated synovitis and H‐type vessels in OA mice, suggesting that PAB may attenuate OA progression by regulating the crosstalk and feedback between pathological synovial inflammation and vessel formation. However, there are some limitations of PAB in clinical application. Systemic administration of PAB or other anti‐angiogenesis drugs may result in some diseases, such as osteoporosis and malnutrition.^48^ Other side effects of PAB drugs may need to be further explored. Therefore, the application of PAB on patients needs more clinical trials, and intra‐articular injection should be administrated rather than systemic treatment.

Paracrine interactions between chondrocytes and macrophages are also of vital during OA.^49^ Importantly, PAB also inhibited chondrocyte catabolism during M1 macrophages polarization (data not shown), while PAB alone could not affect this process under basal conditions in chondrocytes (without stimulus), which indicated that PAB has an anti‐inflammatory effect in inflammatory microenvironment during OA.

Emerging evidences suggested the essential role of NF‐κB signalling in macrophage activation, resulting in exacerbating progression and pathogenesis of OA.^50^, ^51^ Inhibition of NF‐κB activation diminishes IL‐1β‐induced expression of inflammatory mediators and presents a protective effect in both mice and human OA cartilages.^52^ Moreover, NF‐κB signalling participates in LPS‐induced M1 macrophage polarization and iNOS expression.^53^ Here, we defined a critical role of PAB in the regulation of OA progression by inhibiting NF‐κB signalling. Through in vivo and in vitro experiments, we consistently demonstrated that PAB inhibited phosphorylation of p65 and restored the degradation of IκBα. Since PAB ameliorates multiple pathological changes of OA, the regulatory mechanism may be more complex than regulating NF‐κB and mTORC1 signalling pathway, and thus further studies are needed to determine whether PAB modulates OA through other regulatory mechanism.

Several researches revealed that PPARγ prevents OA by associating with mTOR inactivation or promoting demethylation in the articular cartilage.^54^, ^55^ Meanwhile, PPARγ promotes the inactivation of NF‐κB by increasing the expression of PTEN, sirtuin 1 and IκBα in response to inflammation.^36^ In the present study, we identified that PAB inhibited NF‐κB signalling in synovial inflammation by regulating PPARγ. Consistent with previous studies,^6^, ^56^ this study demonstrated that NF‐κB signalling activated in synovial tissues and LPS‐stimulated M1 polarization during OA progression, and PAB treatment not only suppressed NF‐κB signalling by regulating PPARγ expression in vivo and in vitro, but also rescued the phenotypic changes. The associated mechanism of PAB was further confirmed by a specific PPARγ antagonist T0070907 in vitro. However, OA progression may be more complex in vivo, and further studies are needed to detect the effect of PPARγ antagonist in OA mice, but not just in cartilage explants in vitro.

In conclusion, our findings extended the potential clinical applications of PAB. PAB prevents M1 polarization and angiogenesis by stabilizing PPARγ to inhibit NF‐κB signalling, which further attenuates synovial inflammation and OA progression. Hence, our work suggests that targeting synovial inflammation or inhibiting vessel formation at early stage could be an effective therapy for preventing OA and provides potential therapeutic drugs for future anti‐synovitis therapies during OA.

## CONFLICT OF INTEREST

The authors confirm that there are no conflicts of interest.

## AUTHOR CONTRIBUTION

**Xiao Yu:** Data curation (equal); Funding acquisition (supporting); Investigation (lead); Methodology (lead); Project administration (lead); Supervision (lead); Writing‐original draft (lead); Writing‐review & editing (lead). **Jiansen Lu:** Data curation (equal); Investigation (lead); Project administration (equal); Writing‐original draft (equal); Writing‐review & editing (equal). **Hong Guan:** Data curation (lead); Resources (equal); Validation (equal); Writing‐original draft (equal); Writing‐review & editing (supporting). **Dan Wu:** Formal analysis (equal); Software (lead); Validation (lead); Writing‐original draft (equal); Writing‐review & editing (supporting). **Zhiqiang Hu:** Conceptualization (equal); Validation (equal); Writing‐original draft (supporting). **Hongbo Zhang:** Investigation (equal); Methodology (equal); Visualization (equal). **Huaji Jiang:** Conceptualization (equal); Investigation (equal); Methodology (equal). **Jingyao Yu:** Data curation (equal); Visualization (equal); Writing‐review & editing (supporting). **Ke Zeng:** Validation (equal); Visualization (equal). **Hongyu Li:** Methodology (equal); Software (equal). **Haiyan Zhang:** Data curation (lead); Funding acquisition (supporting); Methodology (lead); Resources (lead); Software (equal); Writing‐original draft (equal); Writing‐review & editing (equal). **Chenglong Pan:** Methodology (equal); Resources (equal); Supervision (equal); Writing‐review & editing (equal). **Daozhang Cai:** Conceptualization (equal); Data curation (equal); Funding acquisition (supporting); Methodology (equal); Resources (equal); Validation (equal).

## Supporting information

Supplementary Material

## Data Availability

The data that support the findings of this study are available from the corresponding author upon request.
