# Supplementary material for: Pseudolaric acid B ameliorates synovial inflammation and vessel formation by stabilizing PPARγ to inhibit NF‐κB signalling pathway
Source: J Cell Mol Med. 2021 Jun 11;25(14):6664–78. doi: 10.1111/jcmm.16670 (PMC8278075; doi:10.1111/jcmm.16670)
Supplement: Supplementary file 1 — Supplementary Material [file JCMM-25-6664-s001.docx]

**Additional files1**

**Supplementary Materials and Methods**

**Primary chondrocyte culture**

Tibias were isolated from 8-weeks-old mice. Explants cultured in DMEM/F12 were treated with 10 mM PBS for 1 h, followed by treatment with 50 ng/ml recombinant mouse or human IL-1β in PBS/0.1% BSA, or with PBS/0.1% BSA alone. The explants were harvested and the medium was collected after 3 days culture.

**Co-culture**

Raw 264.7 cells were pretreated with vehicle or PAB (1.5 µM) pretreated for 2 h and then co-treated with LPS (100 ng/mL) for 12 h to induce M1-like macrophages. Supernatants were collected, then co-cultured with ATDC5 for 24 h.

**Supplementary figures**

**Figure S1.**


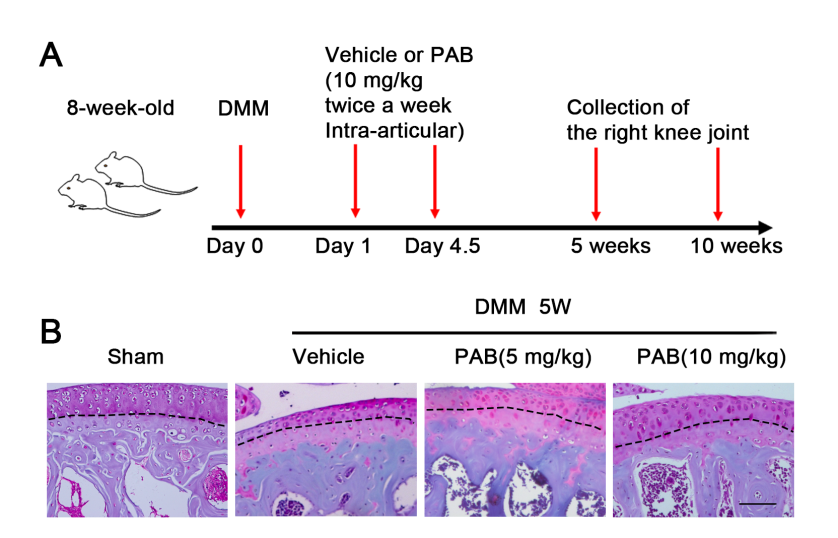


**Figure S1.** Optimal PAB dose screening in DMM mice. (A) Diagram of experiment procedure is shown. The first treatment was started at day 1 after DMM surgery, and the subsequent treatments were administered twice a week for 5, or 10 weeks. (B) Safranin O and Fast Green staining sagittal views of tibial medial cartilage at 5 weeks after DMM surgery. Dotted lines demonstrate tide line. Sham, DMM treated with vehicle, DMM treated with 5 or 10 mg/kg PAB. Scale bar: 50 µm. DMM, destabilizing the medial meniscus; PAB, Pseudolaric acid B; Sham, sham-surgery.

**Figure S2.**


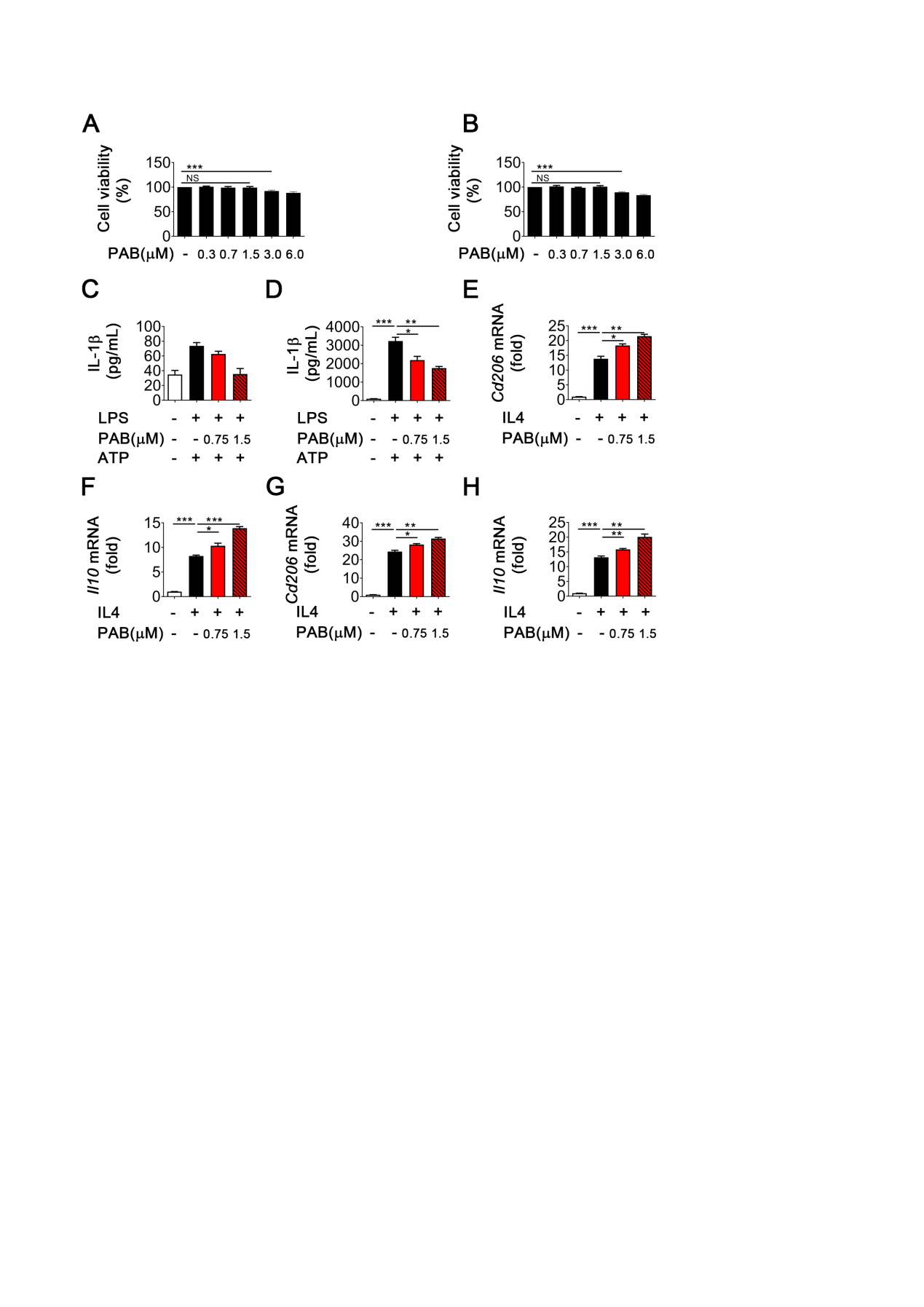


**Figure S2.** PAB effects on cell viability in Raw 264.7 and BMDM cells. (A and B) Raw 264.7 (A) and BMDM (B) cells were cultured with various concentrations of PAB (0–6 µM) for 12 h, and cell viability was evaluated by using Cell Counting Kit-8 assay. (C and D) LPS-induced Raw 264.7 (C) and BMDM (D) cells were treated with ATP for 30 min after co-cultured with PAB for 12 h. Supernatants were collected and subjected to ELISA analysis of IL-1β expression (n=3). (E-H) Quantitative PCR analysis of CD206 and IL-10 in IL-4-induced Raw 264.7 (E and F) and BMDM (G and H) cells treated with or without PAB. **p*<0.05, ***p*<0.01, ****p*<0.001.

**Figure S3.**

**
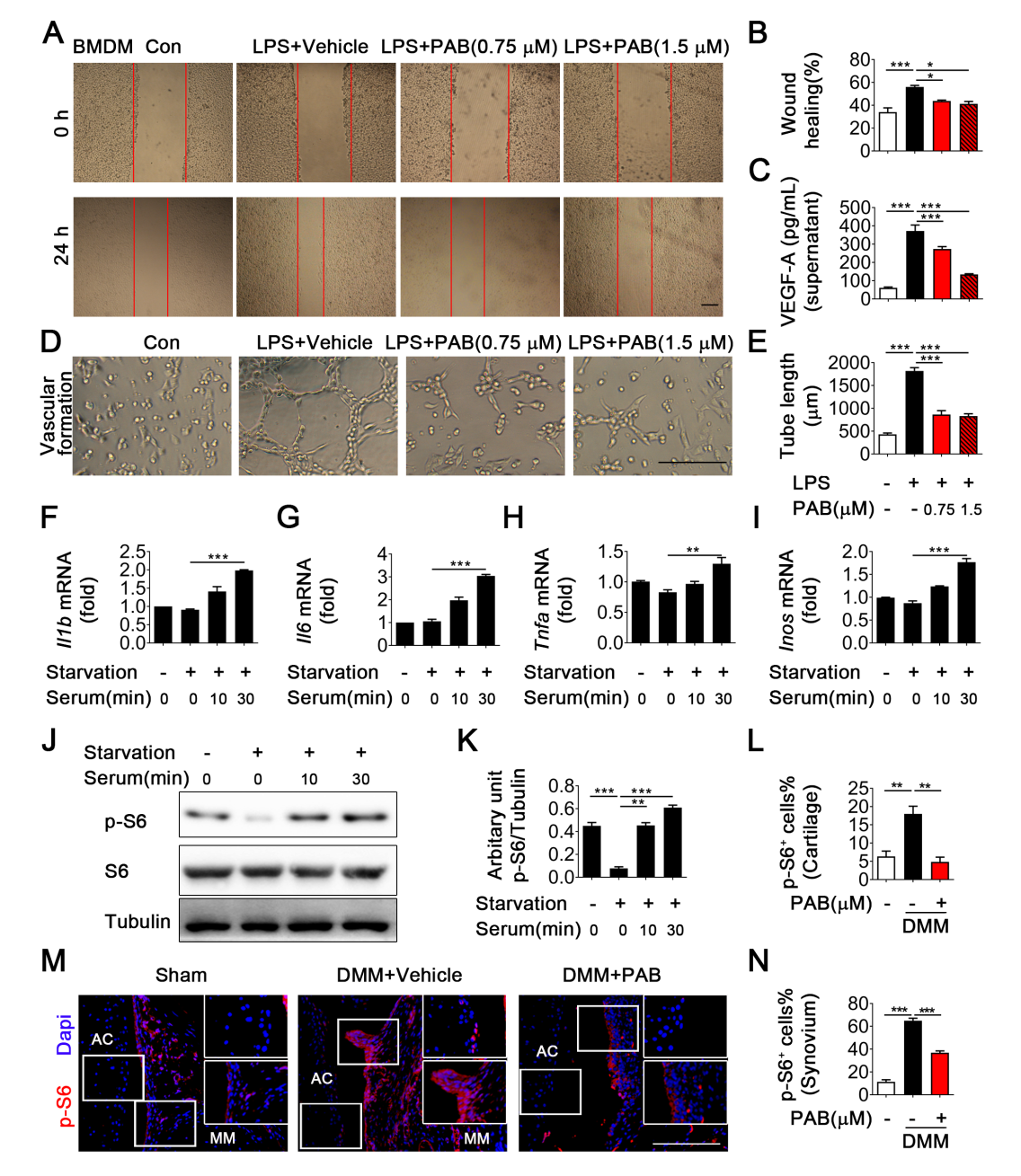
**

**Figure S3.** PAB prevents vessel formation in BMDM and inhibits mTOR signaling. (A-C) HUVECs were co-cultured with CM, vehicle-treated M1 macrophage CM or PAB-treated M1 macrophage CM from BMDM for 24 h. Wound healing assay was performed and shown in (A and B). Scale bar: 25 µm. VEGF-A levels in the supernatant of LPS-induced BMDM cells treated with or without PAB were determined by ELISA (C). (D and E) HUVECs were co-cultured with the supernatant of BMDM as shown, and tube formation was measured by tube formation assay (D). HUVEC tube length was evaluated and shown in (E). (F-K) Upon serum deprivation for 16 h, Raw 264.7 cells were stimulated with DMEM (with or without FBS) for 0, 10, 30 min. Cells were collected and subjected to quantitative PCR analysis of IL-1β (F), IL-6 (G), TNF-α (H) and iNOS (I). p-S6 (S235/236) was determined by immunoblot analysis (J) and analysis of grey intensity was shown in (K). (n=3) (L-N) Immunostaining (L) and quantitative analysis (M and N) of cells positive for p-S6 in vehicle or PAB treated DMM mice at five weeks after surgery. (n=4) Scale bar: 50 µm. Higher magniﬁcation is demonstrated on the right top. DMM, destabilizing the medial meniscus; Sham, sham-surgery; AC, articular cartilage; MM, medial meniscus. **p*<0.05, ***p*<0.01, ****p*<0.001.

**Figure S4.**

**
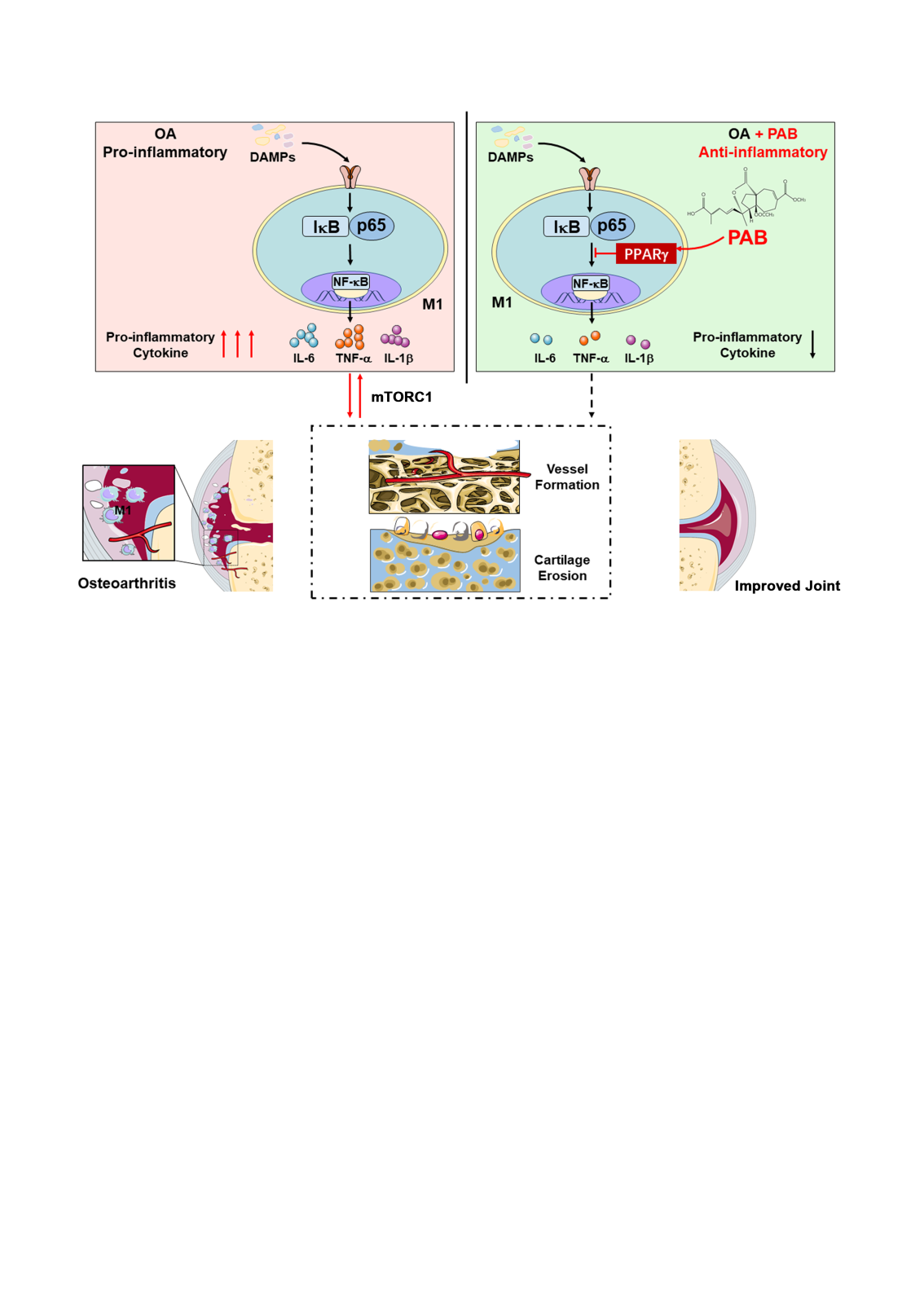
**

**Figure S4.** A schematic model to show that PAB decreases synovial inflammation and vessel formation during OA. During the process of OA, DAMPs activate NF-κB signaling pathway and enhance M1 macrophage polarization, thus releasing mounts of inflammatory cytokines (IL-1β, IL-6 and TNF-α) and accumulating H-type vessels. Treatment of PAB could stabilize PPARγ, thus inhibits NF-κB signaling and M1 macrophage polarization, which further reduces the production of inflammatory cytokines and angiogenesis, leading to amelioration of OA.
